# Affordable mRNA Novel Proteins, Recombinant Protein Conversions, and Biosimilars—Advice to Developers and Regulatory Agencies

**DOI:** 10.3390/biomedicines13010097

**Published:** 2025-01-03

**Authors:** Sarfaraz K. Niazi

**Affiliations:** College of Pharmacy, University of Illinois, Chicago, IL 60612, USA; niazi@niazi.com; Tel.: +1-312-297-0000

**Keywords:** mRNA, recombinant, new drugs, approved functional proteins, mAbs, protein vaccines, biosimilars

## Abstract

mRNA technology can replace the expensive recombinant technology for every type of protein, making biological drugs more affordable. It can also expedite the entry of new biological drugs, and copies of approved mRNA products can be treated as generic or biosimilar products due to their chemical nature. The introduction of hundreds of new protein drugs have been blocked due to the high cost of recombinant development. The low CAPEX and OPEX associated with mRNA technology bring it within the reach of developing countries that are currently deprived of life-saving biological drugs. In this paper, we advise developers to introduce novel proteins and switch recombinant manufacturing to mRNA delivery, and we further advise regulatory authorities to allow for the approval of copies of mRNA products with less testing. We anticipate that mRNA technology will make protein drugs, such as natural and engineered proteins, monoclonal antibodies, and vaccines, accessible to billions of patients worldwide.

## 1. Introduction

Protein drugs comprise therapeutic proteins, functional and engineered monoclonal antibodies, vaccines, and modifications manufactured using a recombinant engineering process that is highly expensive, limiting the availability of hundreds of new protein drugs and keeping the cost of protein drugs high.

The development of mRNA technology has been a decades-long journey, beginning in the 1970s when researchers first proposed that synthetic mRNA could be used to direct protein synthesis within cells. Early studies in 1978 demonstrated that mRNA could be transfected into cells to produce proteins, establishing the feasibility of this therapeutic approach. Significant advancements followed in the 1980s and 1990s with the development of lipid nanoparticles (LNPs), which provided a means to deliver mRNA effectively into cells by protecting it from degradation. A key breakthrough came in 2005, when Katalin Karikó and Drew Weissman incorporated modified nucleosides—such as pseudouridine—into mRNA [1]. This innovation reduced immune activation and improved the stability and translation efficiency of the mRNA, paving the way for its broader application. During the 2010s, preclinical studies demonstrated the potential of mRNA technology for cancer immunotherapy and vaccines targeting infectious diseases.

The COVID-19 pandemic marked a transformative moment for mRNA technology [2], as it propelled the first clinical approvals of mRNA-based products. Pfizer-BioNTech’s Comirnaty (BNT162b2) became the first mRNA vaccine to receive Emergency Use Authorization (EUA) from the U.S. Food and Drug Administration (FDA) on 11 December 2020, followed by full approval on 23 August 2021. Shortly after, Moderna’s Spikevax (mRNA-1273) received EUA on 18 December 2020 [3]. These vaccines, developed at unprecedented speed, highlighted the enormous potential of mRNA technology to address urgent global health challenges. Although mRNA research had been ongoing for decades, these COVID-19 vaccines were the first products to demonstrate its clinical viability, ushering in a new era of vaccine and therapeutic development.

While mRNA can encode short interfering RNAs (siRNAs) or microRNAs (miRNAs) that participate in RNA interference (RNAi) pathways as well (Table 1), their role in expressing proteins is the key focus of this study. Table 1 lists the types of mRNAs that can provide these applications.

## 2. Protein Drugs

The discovery of proteins and the understanding of their structures and functions in the human body emerged gradually over centuries, marked by transformative scientific breakthroughs. The term “protein” itself derives from the Greek word “proteios”, meaning “primary” or “of first importance”, reflecting the early perception that these molecules were fundamental to life. Dutch chemist Gerardus Johannes Mulder is credited with coining the term in 1838 after analyzing organic molecules rich in nitrogen, including substances found in blood and egg whites [9]. This analysis pointed to a new class of compounds distinct from other known organic materials; yet it would take more than a century to uncover the true complexity and significance of proteins.

The structure of proteins was first revealed through pioneering work in the 20th century. The linear sequence of amino acids within a protein, known as its primary structure, was first determined for insulin by Frederick Sanger in 1951 [10]. Sanger’s work was groundbreaking, as it demonstrated that proteins are not just amorphous organic compounds but are composed of specific sequences of amino acids that dictate their function. For this discovery, Sanger was awarded the Nobel Prize in Chemistry, marking a turning point in protein chemistry. This initial success led to further structural discoveries. Linus Pauling and Robert Corey, in the early 1950s, elucidated key structural motifs such as the alpha-helix and beta-sheet [11]. These secondary structures revealed that proteins fold into specific configurations driven by hydrogen bonds—a crucial insight into how proteins achieve their functional forms. In 1958, John Kendrew advanced the understanding of protein structure even further by using X-ray crystallography to solve the three-dimensional structure of myoglobin [12]. This was the first time a protein’s tertiary structure had been visualized at an atomic level, showcasing the intricate folding patterns that are essential for their biological functions.

Simultaneously, the roles of proteins in bodily functions began to unfold. By the mid-20th century, it was well understood that enzymes—specialized proteins—act as catalysts for metabolic reactions, facilitating nearly all chemical processes in the body [13]. The discovery that hormones, antibodies, and transport proteins also consist of proteins underscored their versatility, as these molecules were shown to be integral to signaling pathways, immune responses, and the transport of essential molecules such as oxygen. Through these advancements, proteins came to be viewed as central to nearly every cellular process, and their roles in health and disease became a focal point in medical research [14]. The advent of molecular biology, genomic sequencing, and proteomics in the late 20th and early 21st centuries further solidified proteins as the primary agents of biological function, offering a profound understanding of life at the molecular level and shaping modern medical and biological research.

While significant progress has been made in identifying human proteins, understanding their functions, and linking their deficiencies to diseases, we do not yet have a complete and detailed understanding of all human proteins. The Human Genome Project [15] provided the complete sequence of human DNA, allowing scientists to predict approximately 20,000–25,000 protein-coding genes, thus laying the foundation for protein research. The Human Proteome Project (HPP) [16], an international initiative, aims to map all human proteins, identifying their structures and expression across various body parts. Although we have cataloged most human proteins, databases such as UniProt (https://www.uniprot.org/, accessed on 12 November 2024) and the Human Protein Atlas (https://www.proteinatlas.org/, accessed on 12 November 2024) continue to expand our knowledge, offering detailed information on the functions, structures, and interactions of many proteins.

However, not all essential proteins are “druggable”, meaning they may lack the characteristics that allow for effective modulation through the use of small molecules or biologics. Factors influencing the druggability of proteins include their structure, function, and accessibility within the body. The presence of well-defined binding pockets or cavities that can accommodate drug molecules is crucial; proteins lacking such features are challenging to target with small molecules. Additionally, proteins involved in complex interactions or those without a clear active site may be less amenable to drug targeting. For instance, proteins that function through protein–protein interactions often present flat surfaces, making it difficult for small molecules to bind to them effectively [17].

## 3. Recombinant Technology

Until the arrival of recombinant technology, the only supply of several critical therapeutic proteins was through natural sources. For example, insulin was initially extracted from the pancreas of pigs and cows, and it was widely used for the management of diabetes despite causing immune reactions in some patients due to its animal origin [18]. Human growth hormone (hGH) was sourced from cadaver pituitary glands for the treatment of growth disorders; however, this posed a risk of transmitting prion diseases, such as Creutzfeldt–Jakob disease, due to its sourcing from human tissues [19].

Similarly, Factor VIII and Factor IX—which are crucial for treating hemophilia A and B—were derived from pooled human plasma, presenting a significant risk of contamination with bloodborne viruses such as HIV and hepatitis C [20]. Albumin—a blood volume expander used in cases of hypovolemia and shock—was extracted from pooled human plasma, leading to similar contamination concerns and supply limitations [21]. Immunoglobulins, particularly intravenous immunoglobulin (IVIG), were obtained from large pools of human plasma and are still often sourced in this way, as recombinant versions remain difficult and complex to obtain. Interferons, such as interferon-alpha and interferon-beta, were initially isolated from human leukocytes and fibroblasts for treating viral infections and multiple sclerosis, although in limited quantities due to challenges related to sourcing [22]. Additionally, Follicle-Stimulating Hormone (FSH)—which is used for fertility treatments—was initially purified from the urine of postmenopausal women (urinary FSH) before recombinant FSH became available, which ensured a more stable supply [23]. The hepatitis B vaccine was another key therapeutic originally derived from the plasma of infected individuals, posing a risk of contamination and supply shortages until recombinant versions became available in the early 1980s [24].

Many recombinant proteins are copies of natural functional proteins designed to mimic natural proteins; others are novel or engineered molecules with unique functions distinct from naturally occurring proteins, highlighting the diversity and broad therapeutic applications of biological products. Many therapeutic proteins are constructed, designed, or modified using biotechnological methods to achieve targeted therapeutic effects. Engineered cytokines and growth factors are modified for enhanced stability or extended half-life; such as pegylated interferons used in the treatment of hepatitis, which are attached to polyethylene glycol (PEG) to extend their half-life.

Antibody fragments, such as Fab or scFv, are engineered for specific applications, often with smaller sizes for better tissue penetration. For example, Certolizumab pegol, a PEG-modified antibody fragment against TNF, has been shown to possess improved pharmacokinetics [25], and Blinatumomab (Blincyto) is a bispecific T-cell engager using two scFvs to bridge T cells and cancer cells, facilitating targeted cell lysis in B-cell malignancies [26]. Nanobodies—single-domain antibodies derived from camelids—have been engineered to have only the minimal binding domain, providing high tissue penetration, as seen in the case of Caplacizumab, which is used to treat thrombotic thrombocytopenic purpura [27].

## 4. mRNA Technology

The discovery that ribosomes synthesize proteins evolved through critical research efforts in the mid-20th century, particularly in the 1950s and 1960s. Early work in cell biology had identified ribosomes as small, dense structures within the cytoplasm, yet their function remained unclear. The concept of ribosomes as the site of protein synthesis began to crystallize in the 1950s, as researchers linked ribosomes with protein production in cells [28].

In 1955, cell biologist George Emil Palade observed ribosomes in the rough endoplasmic reticulum through electron microscopy. He noted that ribosomes were frequently associated with newly synthesized proteins, suggesting a possible role in protein assembly. Palade’s observations marked the first significant indication that ribosomes could be involved in protein synthesis. However, it was not until subsequent research—particularly in the early 1960s—that this role was definitively established [29].

The work of François Jacob and Jacques Monod in 1961 supported this by proposing the “messenger RNA hypothesis”. They hypothesized that messenger RNA (mRNA) carried genetic information from DNA to ribosomes, where proteins were synthesized according to this genetic code. Their model predicted that ribosomes and mRNA translate genetic information into functional proteins [30]. Following this, in the early 1960s, studies by Matthew Meselson and Franklin Stahl helped to clarify the mechanics of ribosomal protein synthesis, illustrating the critical role of mRNA as a template [31].

Finally, groundbreaking experiments by researchers such as Marshall Nirenberg and Heinrich Matthaei, who cracked the genetic code in 1961, provided the first concrete experimental evidence that ribosomes use mRNA to assemble amino acids into proteins. By the mid-1960s, these discoveries firmly established ribosomes as the “machines” of protein synthesis, with their role in translating genetic information into proteins being widely accepted in the scientific community [32].

Palade was eventually awarded the Nobel Prize in Physiology or Medicine in 1974 for his contributions to understanding cellular structures and functions, including his discoveries regarding the involvement of ribosomes in protein synthesis [33]. Together, these efforts cemented the role of ribosomes as the sites of protein synthesis—a fundamental insight that revolutionized molecular biology and our understanding of cellular function.

### 4.1. Developing mRNA Protein Delivery

**Figure 1:**
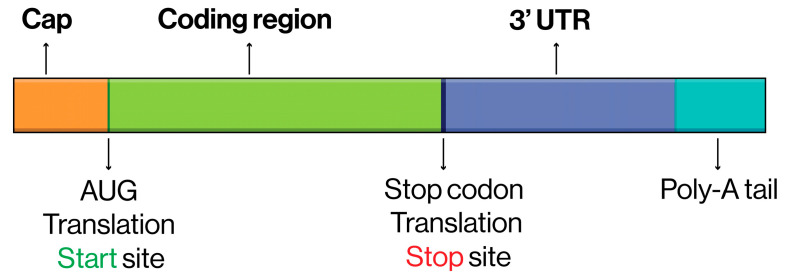
mRNA Structure (Shutterstock Image).

An mRNA therapeutic or vaccine product consists of several carefully designed components, each serving a specific function to ensure stability, efficient translation, and effectiveness. At the 5′ end, the cap structure (a modified guanosine nucleotide, m7G) protects the mRNA from degradation by exonucleases and facilitates ribosome binding for efficient translation initiation. Following the cap, the 5′ untranslated region (5′ UTR) regulates translation efficiency and mRNA stability by interacting with the ribosomal machinery. The coding sequence (CDS), which lies downstream of the 5′ UTR, encodes the protein of interest and begins with a start codon (AUG) and ends with a stop codon (UAA, UAG, or UGA), which signals the termination of translation. Codon optimization within the CDS ensures the use of codons preferred by the target organism to enhance protein synthesis rates. Beyond the coding sequence, the 3′ untranslated region (3′ UTR) plays a critical role in stabilizing the mRNA and modulating its half-life through interactions with regulatory proteins and microRNAs. At the 3′ end, the poly(A) tail—a series of adenine nucleotides—further protects the mRNA from degradation and facilitates interactions with poly(A)-binding proteins, which enhance translation and ribosome recycling. Additionally, modified nucleotides, such as pseudouridine or 1-methylpseudouridine, are often incorporated throughout the mRNA sequence to reduce immune activation, improve stability, and increase translational efficiency. Some mRNA products also include regulatory elements, such as internal ribosome entry sites (IRES) or stabilizing secondary structures, in order to further optimize translation and stability. These sequence components, when combined with a compatible delivery vehicle such as lipid nanoparticles, create a highly adaptable and efficient platform for therapeutic and vaccine applications [34] (Figure 1).

Two principal approaches are employed to cap mRNAs generated through in vitro transcription (IVT). An ARCA (anti-reverse cap analog) can be introduced by substituting the 3′ hydroxyl group of m7G with a methoxy group. ARCA-modified mRNAs typically exhibit elevated translation efficiency and extended half-lives [35,36]. The co-transcriptional trimeric cap analog is the primary capping option, which has been successfully applied in SARS-CoV-2 mRNA vaccines [34].

Modified nucleotides have been employed in mRNA synthesis to diminish the immunogenicity of the mRNA. Examples include 5-methylcytidine (m5C), pseudouridine (Ψ), and N1-methyl pseudouridine (m1Ψ), all of which have been utilized for this purpose [35]. Among these changes, m1Ψ-containing mRNAs have demonstrated a greater capacity for protein synthesis when compared to m5C- and Ψ-containing mRNAs [35]. 

Additional attributes of mRNA have also been shown to influence protein synthesis. Codon usage is crucial in mRNA design, as synonymous codons can influence varying degrees of protein synthesis or impact protein folding and function [35]. Furthermore, additional upstream open reading frames within an mRNA may titrate the translation initiation complex and influence protein translation [35]. 

Enhanced mRNA stability and efficacy can be achieved through multiple methods, including base changes or the application of saRNAs and circRNAs. CircRNAs exhibit greater resistance to exonuclease destruction compared to linear RNAs, as they lack 5′ and 3′ termini [37].

Non-canonical RNA splicing events endogenously produce this type of RNA, and some endogenous circRNAs are known to function as sponges for miRNAs or templates for stress-responsive peptides in mammalian cells [38].

Despite the increasing utilization of saRNAs and circRNAs, both RNA types possess significant drawbacks. For instance, saRNAs are constrained by a potential safety issue, as the alphavirus component may provoke undesirable immune responses; this concern necessitates meticulous consideration in clinical trials. Despite circRNAs exhibiting remarkable intrinsic stability, which can enhance the half-life of mRNA and prolong protein expression, a notable drawback of this method is the intricate manufacturing procedure. In addition to choosing the most advantageous mRNA attributes, the creation of high-efficiency, low-toxicity mRNA vaccines and therapeutics requires the development and optimization of a production process that is capable of consistently producing pure mRNA.

### 4.2. Safety Concerns of mRNA

mRNA-based products, while revolutionary, present several risks that require careful consideration. One significant concern is immunogenicity, as unmodified mRNA can trigger innate immune responses by activating toll-like receptors (TLRs) such as TLR3, TLR7, and TLR8, leading to inflammation and other adverse reactions. Although modifications such as pseudouridine incorporation reduce this risk, improperly optimized sequences can still provoke immune activation [39]. Another challenge is the inherent instability of mRNA, which is prone to rapid degradation by nucleases. While lipid nanoparticles (LNPs) offer some protection, improper handling, or storage—especially outside ultra-cold conditions—can render the product ineffective [40]. Delivery systems such as LNPs, though effective, carry their own risks, including injection site pain, systemic reactions (e.g., fever), and, in rare cases, allergic responses to components such as polyethylene glycol (PEG) [41]. High doses of mRNA may exacerbate inflammatory responses and immune-related side effects, potentially causing tissue damage or autoimmunity [4]. Additionally, improperly designed mRNA sequences may lead to the production of aberrant or misfolded proteins, which can accumulate and cause cellular stress [42].

Long term safety remains an open question, as mRNA-based therapeutics and vaccines are relatively new. Potential risks include prolonged protein expression, chronic inflammation, or unintended effects, although the risk of genomic integration is highly unlikely due to the lack of reverse transcriptase activity in human cells [4]. Manufacturing processes also introduce risks, including contamination with residual solvents or impurities and variability in batch quality, which may provoke adverse reactions or affect efficacy [43]. Despite these risks, advancements in sequence optimization, delivery systems, and manufacturing processes are helping to mitigate these challenges, making mRNA products a promising and adaptable platform for therapeutic and vaccine development.

### 4.3. mRNA Purification and Quality Control

Following synthesis via IVT, an mRNA product may include several contaminants that can facilitate mRNA breakdown. Consequently, various regulatory bodies have developed quality guidelines for mRNA vaccines. In April 2023, the US Pharmacopeia (USP) published the “Analytical Procedures of mRNA Vaccine Quality” to provide Quality by Design (QbD) standards and analytical methodologies for mRNA manufacturing. The elimination of contaminants is an essential phase in mRNA drug development, as mRNA possesses numerous physicochemical characteristics that can be used for purification. For example, mRNA is a large molecule with a molecular weight frequently surpassing 300 kDa and a physical size exceeding 50 nm. These characteristics render the molecule suitable for purification via size exclusion chromatography (SEC). Previously, mRNAs generated via in vitro transcription (IVT) were isolated from the DNA template, enzymes, and surplus nucleoside triphosphates (NTPs) using a Superdex-75 column (Cytiva, Malborough, MA, USA) or alternative size exclusion chromatography (SEC) columns [35]. The conformation of RNA significantly influences the resolution of SEC, and double-stranded RNA (dsRNA) byproducts may be indistinguishable from single-stranded mRNA (ssRNA) due to their comparable sizes. 

However, SEC is generally unsuitable for scaling up in extensive industrial operations. Another characteristic that can be utilized for mRNA purification is the molecule’s strong hydrophobicity [44]. Hydrophobic interaction chromatography (HIC) with appropriate binding salts has been demonstrated to be efficient for separating mRNAs from proteins, dsRNAs, and short RNAs. A frequently employed separation technique in mRNA vaccine synthesis is reverse-phase high-performance liquid chromatography (RP-HPLC). Numerous studies have indicated that RP-HPLC purification can eradicate dsRNA-induced immunity and enhance translatability by a factor of 10 to 1000 relative to non-HPLC-purified mRNAs [37]. Nonetheless, HPLC possesses limitations, including the possible utilization of harmful chemical solvents.

Another method for mRNA purification involves affinity columns, such as oligo-dT columns, which can efficiently exclude contaminants lacking poly(A) tails. This technique has been utilized for SARS-CoV-2 and anti-influenza immunoglobulin G (IgG) mRNAs; however, it is ineffective in adequately distinguishing between ssRNA and dsRNA adequately [45]. An alternate method for eliminating precursor and intron RNAs is affinity purification using highly selective affinity ligands [46]. Due to the challenges in eliminating dsRNA using separation technologies, enzymatic digestion using RNAIII may be necessary, as this enzyme can degrade dsRNA while preserving the integrity of mRNA [45]. Cellulose fibers can alternatively eliminate dsRNAs in an ethanol-containing buffer through particular interactions with 2-hydroxyl residues in the dsRNA. This technology is scalable and has been shown to accomplish 90% removal of dsRNA while ensuring over 65% recovery of mRNA [35]. 

Although contaminants must be eliminated from IVT-generated mRNA, chromatographic purification may influence the structure and biological function of the mRNA. Consequently, the advancement of high-efficiency purification methods remains a continuous endeavor. In this context, employing particular ligands for combinatorial or sequential purification procedures may provide a novel approach to enhance the purity and quantity of IVT-generated mRNAs [47].

### 4.4. Storage and Cold Chain Management

The instability of mRNA-LNPs during storage is mainly due to chemical breakdown induced by hydrolysis and oxidation processes: hydrolysis can cleave phosphodiester links in the mRNA backbone, whereas oxidation may cause base breakage and modifications to the mRNA secondary structure [48]. 

In the lyophilization process, cryoprotective chemicals are essential for averting mechanical breakdown of the mRNA-LNPs caused by ice crystals. The predominant cryoprotectants identified in the literature for freeze-drying microparticles are sugars, including trehalose, sucrose, glucose, and mannitol [49]. Significantly, varying compositions and ratios of LNP components can substantially influence the physical and chemical characteristics of an mRNA-LNP product.

### 4.5. Lipid Compositions and Ligand Targeting

The major challenge of ensuring the safe and efficient delivery of bioactive mRNA that can be easily degraded in the body has led to a significant volume of literature. The delivery mechanisms must guarantee that the mRNA effectively arrives at the target cells and is internalized to elicit the intended therapeutic outcomes. Furthermore, altering the surface of LNP carriers may enable medications to circumvent the immune system and enhance their duration in circulation. PEGylation can enhance the stability of nanoparticles. Restricted immune system activation is essential for the efficacy of mRNA treatments, as undesirable immune responses may result in side effects and diminish the therapeutic effectiveness. Current endeavors are concentrating on creating less immunogenic mRNA sequences and refining LNP delivery technologies to reduce immune responses and enhance cargo expression. The successful advancement of secure and efficacious delivery systems for mRNA-based therapeutics necessitates interdisciplinary research, integrating expertise in the fields of molecular biology, chemistry, materials science, and immunology. Ongoing research is being conducted with the aim of resolving the outstanding obstacles associated with mRNA-based pharmaceuticals in order to fully realize the potential of this novel category of therapeutics. 

At present, all FDA-sanctioned LNPs consist of four categories of lipids: ionizable lipids, phospholipids, cholesterol, and PEG lipids [50]. The use of PEG lipids is concerning, as they may provoke the generation of anti-PEG antibodies. Repeated administration of PEG-containing mRNA-LNPs can elicit anti-PEG antibodies that target PEG-coated mRNA-LNPs, thereby diminishing their transportation efficiency [51]. While substantial safety concerns regarding mRNA vaccines have not emerged, it is crucial to acknowledge that their clinical application remains relatively novel, necessitating further investigation into their side effects and other constraints.

The swift advancement and notable effectiveness of mRNA-based COVID-19 vaccines have generated significant interest in mRNA-based therapeutics or vaccinations for numerous viral and immunological disorders. The chemical synthesis of stable mRNA was a significant advancement that broadened the drug development capabilities of mRNA technology. Due to the ability of IVT-generated mRNAs to be directly translated into therapeutic proteins, mRNA is regarded as a highly promising therapeutic method within the pharmaceutical sector. The potential of mRNA-based therapeutics extends beyond vaccinations for infectious disorders; in particular, this technology may potentially serve as an effective platform for gene and protein therapy. One benefit of mRNA-based therapeutics is their immunity to the elevated production expenses linked to antibody-based medications. Another advantage is that improved delivery systems may enhance the treatment efficiency through enabling the targeted distribution of therapeutic nucleic acids to specific cells [52]. 

LNPs are effectively internalized by cells and delivered to endosomal compartments. To effectively transport the mRNA payload into the cytosolic area, the LNPs must extricate themselves from endosomes. In the acidic endosomal milieu, the ionizable lipids in lipid nanoparticles (LNPs) are protonated, acquiring a positive charge that facilitates their binding to negatively charged lipid molecules on the endosomal membrane. This connection initiates phase transition and fusion of the lipid nanoparticle with the endosomal membrane, thereby releasing the nucleic acid payload into the cytoplasm. A major constraint of this method is that the majority of endocytosed LNPs are ultimately sent to lysosomes for destruction, with only a minuscule fraction (about 2%) effectively evading endosomes to deliver the nucleic acid payload. Multiple approaches may address this constraint, such as the creation of innovative ionizable lipids, the integration of auxiliary lipids, and the addition of other unique materials. Researchers anticipate that these methods will improve the endosomal escape of lipid nanoparticles (LNPs) and augment the efficacy of nucleic acid delivery via LNPs.

## 5. Recombinant vs. Ribosomal

The FDA’s drug approval process is comprehensive and can span several years. In total, bringing a new drug to market can take approximately 10 to 15 years. Using mRNA technology instead of recombinant protein methods can significantly reduce drug development timelines for several reasons:

- Eliminating protein production: Traditional recombinant protein production requires creating, optimizing, and scaling up cell lines to produce the protein. mRNA eliminates this process, as mRNA directly encodes the protein of interest, which is then made within the patient’s cells. This reduces both preclinical and manufacturing timelines.
- Simplified manufacturing: mRNA synthesis is more straightforward and faster than protein production, as it involves in vitro transcription, which can be completed within weeks. In contrast, recombinant proteins require complex bioreactors, extensive purification, and scaling-up processes, which can take months or even years.
- Rapid design and iteration: mRNA sequences can be quickly modified if adjustments are needed, such as changes in dose or specific protein regions. This adaptability is especially valuable in the early development phase, allowing researchers to iterate quickly without lengthy cell line adjustments.
- Streamlined preclinical testing: mRNA drugs (mainly vaccines) may bypass some traditional preclinical studies, as they have a different safety profile than recombinant proteins. The risk of toxicity is generally lower, as mRNA does not integrate into the genome and is rapidly degraded after the protein is expressed.
- Potential for faster clinical trials: mRNA’s faster production enables quicker scaling-up for clinical trials, reducing the wait time between phases. mRNA therapies also tend to elicit a robust immune response, which may shorten dose-ranging and efficacy assessment stages, especially in vaccines.
- Regulatory pathways and accelerated approval: The success of COVID-19 mRNA vaccines has led to new regulatory insights and expedited review pathways, potentially offering shorter approval timelines for future mRNA-based therapeutics.
- Estimated time reduction: mRNA technology can potentially reduce the typical drug development timeline from around 10–15 years to as short as 5–8 years in some cases, depending on the disease and the regulatory pathway chosen.

While mRNA technology can significantly reduce costs in several areas, the reduction is often not strictly proportional to the time savings. mRNA can impact expenses compared to traditional recombinant protein-based drugs in the following ways:

- Lower manufacturing costs: mRNA production uses more straightforward and more scalable processes. Traditional recombinant proteins require costly mammalian or microbial cell culture systems, which are resource-intensive and involve complex purification steps. In contrast, mRNA synthesis can be performed rapidly in vitro, cutting manufacturing costs by approximately 30–40%.
- Reduced infrastructure and facility equirements: Recombinant protein production often demands specialized bioreactors, sterile environments, and stringent quality controls. In contrast, mRNA manufacturing can use more straightforward equipment and smaller facilities, reducing the fixed costs and capital investment needed for facilities.
- Streamlined preclinical and clinical phases: mRNA’s flexibility reduces the need for extended preclinical testing and enables a faster transition through clinical trials. This can lower costs associated with maintaining and managing trials, reducing expenses for staffing, data monitoring, and patient recruitment.
- Less batch variability and simplified quality control: Each batch is less variable, as mRNA is obtained through a predictable in vitro transcription process. This contrasts with protein products, which can vary in a manner depending on cell line behavior and production conditions. Reduced variability translates to less waste and fewer quality control expenditures.
- Reduced R&D costs through rapid iteration: As mRNA sequences are easily adjustable, early-stage development costs are decreased as researchers can adjust sequences without creating new cell lines. This makes R&D less expensive—especially in preclinical phases—as modifications to doses or protein regions do not involve extensive re-optimization.

Overall, these factors can reduce the cost of developing an mRNA-based therapeutic to potentially half or less of that of a traditional protein-based therapeutic, depending on the product’s complexity and regulatory requirements. While some estimates suggest around a 50% reduction in expenses, the savings will depend on the production scale and regulatory complexities associated with each specific mRNA application [53].

Using mRNA for novel monoclonal antibodies (mAbs) can potentially be even faster and more cost-effective than traditional recombinant methods for mAb development and production; however, there are specific considerations:

- Faster development timeline: Producing mAbs typically involves generating stable cell lines, optimizing them, and scaling production. This process can take years. With mRNA, the sequence coding for the mAb can be synthesized and delivered directly to the body’s cells, which then produce the antibody. This approach skips cell line development, reducing early-stage development by up to 50%. Additionally, mRNA-encoding mAbs can be optimized and modified more rapidly than protein-based mAbs, allowing for faster adjustments and fewer delays if dose modifications or structural changes are needed.
- Lower production costs: Traditional mAb production is complex, requiring costly cell culture facilities, extensive purification processes, and quality control. mRNA bypasses these steps, as manufacturing involves producing mRNA, which is more straightforward and can be performed in smaller facilities. mRNA is highly scalable, allowing manufacturers to adjust production quickly to meet demand without needing major infrastructure expansion, as required for conventional mAb production, which is costly. This reduces batch production costs significantly. Producing mRNA with consistent quality is often simpler than ensuring consistency in mAb production across batches, especially when using complex systems such as CHO cells.
- Reduced R&D costs: The mRNA platform is highly adaptable, allowing for rapid iteration at a lower cost if changes are required. For mAbs, adjustments to the mRNA sequence do not require creating new cell lines, thus eliminating the associated time and expense. As mRNA enables in vivo expression of mAbs, early testing can begin faster and at potentially lower costs.
- Speed of regulatory approval: The FDA has become increasingly familiar with mRNA technology, thanks to recent approvals and accelerated pathways. For mAb-based treatments, this familiarity could result in quicker regulatory processes if the safety profiles are favorable, although this is still a developing area. For novel mAbs, the use of mRNA can cut development costs and timelines even more than typical mRNA-based proteins. Costs could be reduced by over half, compared to conventional mAb production methods, with timelines potentially shortened from 8–12 years to as little as 4–7 years for specific applications, especially if regulatory bodies offer expedited paths for mRNA-based mAbs.

## 6. New Protein Drugs

While knowledge about endogenous proteins is well developed, only a tiny fraction of these proteins has been developed to fulfill their deficiency or use them for a defined novel treatment, as the high cost of recombinant manufacturing does not justify their development due to their relatively small market. These constraints can be removed if the primary method of developing proteins shifts to mRNA delivery, which will allow for remarkable opportunities such as those associated with the following examples:

- Adipocyte Fatty Acid-Binding Protein (AFABP) transports fatty acids within adipose tissue. It may help to regulate fat metabolism, with targeting AFABP having been proposed as a potential strategy to reduce fat storage and enhance fat mobilization [48].
- AMP-Activated Protein Kinase (AMPK) is an energy-sensing enzyme that promotes autophagy, reduces inflammation, and prevents age-related diseases; it can be activated through diet, exercise, or compounds such as metformin [54].
- Amyloid Precursor Protein (APP) is essential for neuronal health, though improper processing can lead to Alzheimer’s disease; maintaining normal APP levels while preventing harmful byproducts has been a focus of research [55].
- Brain-derived Neurotrophic Factor (BDNF) is crucial for synaptic plasticity, and its deficiency has been linked to learning deficits and cognitive decline, with therapeutic approaches aimed at increasing BDNF levels showing promise. Nerve Growth Factor (NGF) supports neuronal survival, with NGF gene therapy having been explored for Alzheimer’s disease [56].
- Calcium/Calmodulin-Dependent Protein Kinase II (CaMKII) is essential for memory processes and targeting CaMKII signaling through therapy may support cognition. CREB (cAMP Response Element-Binding Protein) regulates memory consolidation, and therapies enhancing CREB may benefit learning in patients with neurodegenerative conditions [57].
- Collagen is a structural protein in connective tissues. Its extensive post-translational modifications—such as hydroxylation and glycosylation—are challenging to replicate in recombinant systems. While recombinant collagen-like proteins have been produced, they may not fully mimic the properties of native collagen [52].
- Elastin provides elasticity in tissues such as skin and blood vessels. Recombinant production of elastin is complicated by its repetitive sequences and cross-linking requirements. Some elastin-like polypeptides have been synthesized recombinantly but they may not fully replicate the properties of native elastin [58].
- Fibroblast Growth Factor (FGF) supports tissue repair, blood vessel formation, and cell survival in heart tissue [59].
- Forkhead Box O (FOXO) proteins, such as FOXO3, regulate cell survival, stress resistance, and metabolism, while enhancing antioxidant defenses and DNA repair, with FOXO3 being associated with longer lifespans [60].
- Fragile X Mental Retardation Protein (FMRP) is essential for cognitive development, with therapies targeting its affected pathways in patients with intellectual disabilities [61].
- Heat Shock Proteins (HSPs), particularly HSP70, protect other proteins from damage by refolding misfolded proteins, preventing the aggregation associated with Alzheimer’s [62].
- Irisin—a protein produced by muscles during exercise—is thought to help convert white fat (which stores energy) into brown fat (which burns energy) in a process called “browning”, potentially increasing energy expenditure and reducing fat stores [63].
- Keratin is a structural protein in the hair, nails, and skin. Its insolubility and tendency to form strong disulfide bonds make its recombinant production challenging. Recombinant keratin-like proteins have been developed, but they may not fully replicate the characteristics of native keratin [64].
- Klotho—a protein associated with lifespan extension—regulates calcium and phosphate metabolism, supports kidney and cardiovascular health, and reduces inflammation, with higher levels having been linked to improved brain function [65].
- Leptin is a hormone that regulates energy balance by signaling the brain to reduce appetite when fat stores are adequate, potentially promoting fat burning in individuals with leptin sensitivity [66].
- Lipoprotein Lipase (LPL) breaks down triglycerides in the bloodstream into free fatty acids, which can be used for energy or stored in fat cells [67].
- Myosin is a motor protein complex that is essential for muscle contraction. Its large size and complex assembly make its recombinant production challenging. While some subunits or fragments are available, full-length functional myosin is less commonly produced recombinantly [68].
- NAD+ is used for cellular energy production and repair, with NAD+ levels declining with age [69].
- Neuregulin-1—which is essential for brain development—supports cognitive health, particularly in patients with schizophrenia [70].
- Oxytocin receptors influence social learning, and oxytocin treatments have been explored in the context of autism [71].
- p53—known as the “guardian of the genome”—prevents DNA damage through regulating the cell cycle and apoptosis, reducing cancer risk and supporting longevity [72].
- Platelet-Derived Growth Factor (PDGF) recruits fibroblasts and smooth muscle cells for wound healing and scar formation [73].
- Reelin—which is vital for brain development—has been linked to autism and schizophrenia, with modulation of its signaling considered as a potential treatment [74].
- SIRT1 activates pathways for stress protection, DNA repair, and antioxidant defenses, while SIRT3 (located in the mitochondria) regulates energy production and protects against oxidative stress [75].
- Synaptic proteins such as PSD-95 and Synapsin are fundamental to memory, while GABA and glutamate receptor imbalances have been related to intellectual disabilities and neurological disorders [76].
- Tubulin forms microtubules and is crucial for cell structure and division. Recombinant expression of tubulin is complex due to its tendency to form aggregates and the need for specific chaperones for proper folding. Native tubulin is often purified from natural sources for research purposes [77].
- Vascular endothelial growth factor (VEGF) promotes nutrient and oxygen supply and is crucial for healing, especially in the context of muscle, bone, or skin injuries [78].

### Regulatory Guidelines

The FDA includes certain RNA-based products under its gene therapy regulatory framework—mainly when they modify or regulate gene expression within cells to produce a therapeutic effect. Some of the RNA products that the FDA considers as gene therapies are detailed below. How the FDA and EMA classify mRNA is a complex topic [79], which is evolving as many mRNA products besides vaccines are emerging, such as those designed to express therapeutic proteins. Early clinical trials of mRNA therapeutics include studies of paracrine vascular endothelial growth factor (VEGF) mRNA for heart failure and CRISPR-Cas9 mRNA for a congenital liver-specific storage disease. However, many challenges remain to be addressed before mRNA can be established as a general therapeutic modality with broad relevance to rare and common diseases. Various new technologies are being developed to surmount these challenges, including approaches to optimize mRNA cargos, lipid carriers with inherent tissue tropism, and in vivo percutaneous delivery systems. The judicious integration of these advances may unlock the promise of biologically targeted mRNA therapeutics—beyond vaccines and other immunostimulatory agents—for the treatment of diverse clinical indications [80]. In this study, we describe the anticipated new types of mRNA products.

Initially, neither the FDA nor the EMA had specific regulations for mRNA-based therapeutics as vaccines. Consequently, during the COVID-19 pandemic, regulatory agencies resorted to an emergency “rolling review” process, which allowed for data submission and review in real-time, thus expediting the vaccine approval process. This emergency review, while responsive, omitted some standard tests required for GTPs, raising questions about long term safety. This rapid approval process has left substantial gaps in the study of the pharmacokinetics, toxicology, and long term biodistribution of these vaccines—areas typically scrutinized in gene therapy evaluations. These gaps are relevant, as mRNA vaccine developers aim to broaden their application to non-pandemic uses, such as influenza vaccines.

## 7. Recombinant Switchover

Using mRNA to deliver a protein that has been previously approved as a recombinant protein involves several key steps and considerations to ensure effective translation and bioavailability.

- Designing the mRNA sequence for the biosimilar protein and sequence fidelity: The mRNA sequence should encode the exact amino acid sequence of the approved biosimilar protein. This requires designing the mRNA with codons optimized for efficient translation in the target cells (e.g., human cells), which helps to achieve high expression levels [81].
- 5′ and 3′ untranslated regions (UTRs): Optimized UTRs can enhance the stability and translational efficiency of mRNA. Choosing UTRs that increase translation in specific tissues or cells can help to achieve targeted protein delivery. Codon-optimized mRNA constructs have demonstrated significantly improved translation rates for proteins such as erythropoietin, which is commonly delivered as a recombinant protein.
- Modified nucleosides: Incorporating modified nucleosides (e.g., pseudouridine and 1-methyl pseudouridine) into the mRNA can reduce immune recognition while increasing its stability and translational efficiency [1].
- Poly(A) tail and cap structure: A cap structure (e.g., Cap 1) and a poly(A) tail increase the stability and translation of mRNA, enhancing the duration and consistency of protein production.
- LNP formulation: Encapsulating mRNA in lipid nanoparticles (LNPs) protects it from degradation in the bloodstream, facilitates cellular uptake, and promotes endosomal escape, enabling the mRNA to reach the cytoplasm, where it can successfully be translated into the desired protein [41].
- Demonstrating bio similarity: To meet bio similarity standards, the mRNA-produced protein must demonstrate similarity in structure, function, and therapeutic efficacy to the original recombinant protein. This often involves comparative testing, including in vitro and in vivo assays for pharmacokinetics, pharmacodynamics, immunogenicity, and stability.
- FDA and EMA requirements: Regulatory agencies require evidence that the mRNA-delivered protein matches the reference product regarding its potency and safety, which may include animal studies and clinical trials.
- Applications: mRNA delivery is under investigation for therapeutic proteins that traditionally require regular injections, such as monoclonal antibodies, clotting factors, and enzymes for genetic diseases. This approach aims to enable endogenous protein production, potentially reducing the required dosing frequency.
- Clinical example: mRNA-based delivery of Factor VIII for hemophilia has been studied as a long-lasting alternative to recombinant protein infusions [82,83]. This approach to delivering biosimilar proteins via mRNA is in its early stages, but it holds promise for making therapeutic protein delivery more effective and patient friendly. The development process includes complex regulatory and manufacturing considerations in order to ensure that the therapeutic effect and safety of the mRNA-delivered protein match those of the approved recombinant versions.

This structured, multi-faceted approach ensures that an mRNA product is rigorously evaluated, combining relevant aspects from biosimilar guidelines with additional mRNA-specific testing. Through confirming its equivalency in sequence, structure, stability, function, and immunogenicity, this guideline ensures that an mRNA product can be reliably positioned as a biosimilar.

The FDA has approved 100 therapeutic proteins [84], 144 monoclonal antibodies, and 29 recombinant protein vaccines [84]. More than 200 of these products are now off-patent.

As of 2024, the global market for therapeutic proteins has been estimated to increase from USD 132.4 billion in 2023 to USD 203.6 billion by 2029, with a compound annual growth rate (CAGR) of 7.5% from 2024 through 2029 [85]. Their affordability is best viewed in terms of the reimbursements made by the CMS for Medicare patients, which are the lowest price paid for drugs: the per gram cost is over USD 25 million for Inotuzumab ozogamicin, while that for the same drug with a different antibody is USD 2.7 million. The lowest-cost antibody is about USD 350,000 per gram, while the manufacturing cost for these products is generally less than USD 100 per gram [86].

Recombinant vaccines have recently risen and include their approval date in parentheses: Measles, Mumps, and Rubella Virus Vaccine Live (1978); Rabies Vaccine (1980); hepatitis B Vaccine (1986); Haemophilus b Conjugate Vaccine (1989); Typhoid Vi Polysaccharide Vaccine (1994); Varicella Virus Vaccine Live (1995); Anthrax Vaccine Adsorbed (2002); Tetanus and Diphtheria Toxoids Adsorbed (2003); Tetanus Toxoid, Reduced Diphtheria Toxoid, and Acellular Pertussis Vaccine, Adsorbed (2005); Measles, Mumps, Rubella, and Varicella Virus Vaccine Live (2005); Human Papillomavirus Vaccine (2006); Smallpox (Vaccinia) Vaccine, Live (2007); Rotavirus Vaccine, Live, Oral (2008); Japanese Encephalitis Vaccine, Inactivated (2009); Pneumococcal 13-valent Conjugate Vaccine (2010); Adenovirus Type 4 and Type 7 Vaccine, Live, Oral (2011); Influenza Vaccine (2013); Meningococcal Group B Vaccine (2015); Cholera Vaccine, Live, Oral (2016); Zoster Vaccine Recombinant, Adjuvanted (2017); Dengue Tetravalent Vaccine, Live (2019); Smallpox and Monkeypox Vaccine, Live, Non-Replicating (2019); Ebola Zaire Vaccine, Live (2019); Pneumococcal 20-valent Conjugate Vaccine (2021); Pneumococcal 15-valent Conjugate Vaccine (2021); Tick-Borne Encephalitis Vaccine (2021); COVID-19 Vaccine (Recombinant, Adjuvanted) (2022); Respiratory Syncytial Virus (RSV) Vaccine (2023); and Respiratory Syncytial Virus (RSV) Vaccine (2023).

### Development Guideline

There are no regulatory guidelines addressing the development of new mRNA products that are copies of approved recombinant drugs, and the FDA and EMA treat these products as new drugs or biologics. However, several options are available to developers to reduce the testing burden under the FDA’s GASK guideline [87], which allows the developers to present scientific arguments to resolve issues with better efficiency and rationality.

Animal toxicology studies will be required to establish a product’s safety if using an LNP formulation; if the developer uses a composition that the FDA has approved, the extent of non-clinical studies will be reduced but not removed, as proof of safety is required. Regardless of whether only the subcutaneous route is used for administration of the product, intravenous research will also be necessary. Table 2 details a proposed animal toxicology testing plan.

- Protein yield predictions derived from animal expression research are deemed appropriate when utilizing rodent species, as their expression levels typically surpass those observed in humans. A single mRNA molecule can produce hundreds or even thousands of protein molecules, contingent upon the stability and functionality of the mRNA. An initial phase 0 investigation in humans will be necessary to validate the mRNA dose–protein yield profile, according to animal conversion results.
- A short phase II study with 4–6 subjects allow for the establishment of a dose–response relationship and the pharmacokinetic disposition profile. These profiles are expected to not match well, due to the inevitable differences in the absorption half-life and yield. However, the FDA requires at least a comparison of the total AUC to relate it to the total dose reaching the body. Notably, the developer requests the same indications, requiring this equivalence to be established.
- A phase III or comparative efficacy study will likely not be required; however, this waiver will require the developer to present a scientific argument to the FDA.
- The FDA will approve the plasmid design and construction, with relevant tests potentially including those shown in Table 3.

**Table 3:** Analytical methods for plasmid testing.

Test	Method	Specification
Identity	Restriction Enzyme Analysis with Agarose Gel Electrophoresis	Consistent with the reference standard
Plasmid Identity	Sanger Sequencing	Matches reference standard
Purity	A260/A280	1.8–2.0
Concentration	A260	Default 1 mg/mL
Residual Host Cell Protein	HCP ELISA	<1%
Residual Host Genomic DNA	Quantitative PCR	<5%
Residual Host RNA	Agarose Gel Electrophoresis	Non-detectable at 200 ng
Endotoxin	Quantitative LAL assay	<0.01 EU/µg
Bioburden	USP <61>	No growth after 48 h

The CMC data requirements are well defined and should be complied with. Notably, as the mRNA is a chemical entity, little batch-to-batch variability will be allowed. Furthermore, unlike with proteins, there are no post-translational concerns. Table 4 and Table 5 list the release specification methods for DS and DP that are considered acceptable by the FDA.

## 8. Biosimilar mRNA

The biological drug category of biosimilars involves only therapeutic proteins and not recombinant vaccines approved as biological products. Therefore, mRNA products will not qualify as biosimilars under the current definition of biosimilars. On the other hand, an mRNA product is a chemical product that can be copied exactly as a reference product, better than that which is possible for proteins with many structural variabilities (e.g., post-translational modifications, none of which apply to mRNA products). No guidelines are available from the FDA or EMA for the development of these products. However, a developer can take advantage of scientific arguments, as presented below, in order to secure many concessions in the testing process; even though the title of “biosimilar” is not likely to be given, neither would be the title of a generic drug.

As of November 2024, the FDA has approved the following mRNA products:

- Comirnaty (BNT162b2), developed by Pfizer-BioNTech, became the first mRNA vaccine approved for COVID-19 prevention in August 2021, marking a significant milestone in mRNA vaccine technology with FDA approval. Shortly after, Spikevax (mRNA-1273) by Moderna was approved in December 2020 as the second mRNA vaccine for COVID-19 prevention, further solidifying the role of mRNA vaccines in the pandemic response. In May 2024, Moderna also introduced mRESVIA (mRNA-1345), the first mRNA vaccine approved specifically for preventing Respiratory Syncytial Virus (RSV) in adults aged 60 and over, expanding mRNA applications beyond COVID-19 [88].

Several biotechnology companies have publicly disclosed their mRNA-based products that are currently under development. These products span various therapeutic areas, including infectious diseases, oncology, and rare genetic disorders. An overview of some essential mRNA products in development is provided below:

- Moderna, Inc. has a robust pipeline of mRNA-based vaccines and therapeutics across multiple areas. For infectious diseases, it includes the approved COVID-19 vaccine (mRNA-1273), marketed as Spikevax^®^, a next-generation COVID-19 vaccine (mRNA-1283) designed for more accessible storage and administration, and seasonal influenza vaccines (mRNA-1010, mRNA-1020, mRNA-1030) targeting multiple influenza strains. Moderna is also developing an RSV vaccine (mRNA-1345) for older adults and combination vaccines, such as a flu and COVID-19 vaccine (mRNA-1083) and a triple vaccine targeting flu, COVID-19, and RSV (mRNA-1230). In the oncological field, Moderna has collaborated with Merck for the development of Individualized Neoantigen Therapy (INT) (mRNA-4157)—a personalized cancer vaccine targeting melanoma and other cancers. Additionally, for rare diseases, Moderna is developing therapies for Propionic Acidemia (mRNA-3927) and Methylmalonic Acidemia (mRNA-3705) (www.moderna.com).
- BioNTech SE—widely recognized for its COVID-19 vaccine developed with Pfizer—is advancing its pipeline with mRNA therapies for infectious diseases, such as the influenza vaccine BNT161 and a shingles vaccine (BNT163), also developed in conjunction with Pfizer. BioNTech’s oncology portfolio includes FixVac (BNT111), an mRNA vaccine targeting advanced melanoma, and the Individualized Neoantigen Specific Immunotherapy (iNeST) (BNT122), developed in collaboration with Genentech (www.biointech.com).
- CureVac N.V. is developing mRNA-based vaccines and therapeutics, including a second-generation COVID-19 vaccine (CVnCoV) and a rabies vaccine (CV7202) for infectious diseases. It is also exploring mRNA-based cancer vaccines targeting various tumors (www.curevac.com).
- Translate Bio (now part of Sanofi) focuses on mRNA therapeutics, including influenza and COVID-19 vaccines and an inhaled mRNA therapy for cystic fibrosis (MRT5005) (https://www.sanofi.com/en).
- Arcturus Therapeutics is developing self-amplifying mRNA vaccines, such as a COVID-19 vaccine (ARCT-021) and an influenza vaccine, as well as an mRNA therapy for Ornithine Transcarbamylase (OTC) Deficiency (ARCT-810), a urea cycle disorder (https://arcturusrx.com).
- Gritstone bio is working on mRNA-based cancer immunotherapies, including Granite (SLATE), personalized immunotherapy for solid tumors, and the CORAL program, which is focused on the development of mRNA vaccines for infectious diseases, including COVID-19 (https://gritstonebio.com).
- eTheRNA Immunotherapies focuses on mRNA-based immunotherapies, specifically, TriMix-Based Cancer Vaccines designed to stimulate immune responses against tumors (https://www.etherna.be).
- Imperial College London is advancing a self-amplifying RNA (saRNA) vaccine platform, including a saRNA COVID-19 vaccine targeting SARS-CoV-2 (https://www.imperial.ac.uk).
- Chimeron Bio is developing mRNA therapies with OncoRNA to target solid tumors, as well as various infectious disease vaccines.
- Providence Therapeutics is advancing mRNA vaccines, including PTX-COVID19-B—an mRNA COVID-19 vaccine candidate targeting SARS-CoV-2—and personalized cancer vaccines (https://www.chimeron.com).

### 8.1. Scenario 1

If the mRNA sequence of a new product is identical to that of an already approved mRNA product—meaning it has the same nucleotide sequence, codon optimization, and untranslated regions (UTRs)—then it is expected to yield the same protein with identical primary and secondary structures. This is because the mRNA’s codon sequence and subsequent protein synthesis dictate the primary structure (amino acid sequence) and secondary structure (local folding; e.g., alpha helices and beta sheets) of the resultant proteins.

Furthermore, if both products use an identical lipid nanoparticle (LNP) formulation, the pharmacokinetics and biodistribution are expected to be similar, assuming similar administration routes and dosages. The LNPs, being the delivery vehicles, are critical to protecting the mRNA, aiding in cellular uptake, and modulating the immune response. When the composition, size, and charge of LNPs are identical, they should theoretically present similar distribution and cell targeting profiles.

The main difference between the two products would likely be yield variations, potentially due to slight differences in the manufacturing process or conditions, which could impact the efficiency of mRNA encapsulation or stability. These differences might affect the dose required to achieve a therapeutic level. Still, they should not lead to differences in the protein’s structure or the safety profile, assuming that the mRNA and LNP are identical.

However, if a pharmacokinetic (PK) comparison between the original and the new mRNA product demonstrates an identical profile, then any concerns regarding differences in yield effectively become irrelevant. Identical PK profiles suggest that both products achieve comparable concentrations, distribution, metabolism, and elimination in the body, leading to similar therapeutic levels over time.

This alignment in PK would indicate that any variations in manufacturing yields do not impact the ultimate bioavailability or effectiveness of the product. Therefore, with identical mRNA sequences and LNP formulations yielding the same PK profiles, there would be no reason to expect differences in safety, efficacy, or dosing requirements between the original product and the new one. Thus, the dosing of the biosimilar product can be the same as that of its reference products.

### 8.2. Scenario 2

If the mRNA products have identical PK profiles but differ in their untranslated regions (UTRs) or LNP formulations, they could be considered functionally equivalent concerning the therapeutic protein’s bioavailability and overall exposure. However, these differences could still have influences on immunogenicity or other biological interactions, as follows:

mRNA Sequence Variations (in UTRs): Changes in UTRs can affect the efficiency of translation and stability, potentially leading to different protein expression rates or half-lives at the cellular level. Despite an identical PK profile at the systemic level, these differences could subtly influence intracellular dynamics, possibly impacting protein synthesis in specific tissues.

LNP Formulation Differences: LNPs can influence the immune response, biodistribution to specific tissues, and the cellular uptake of mRNA. Different LNP compositions may lead to distinct immunogenic profiles, even under identical PK profiles. Immune activation varies based on LNP characteristics such as lipid types, particle size, and charge, which could elicit varied innate or adaptive immune responses. This may affect their tolerability, mainly if the immune system responds differently to the LNP components.

In regulatory terms, if two products have identical PK profiles and demonstrate no significant immunogenic differences in clinical studies, they might be considered equivalent from a therapeutic standpoint. However, the evaluation would still require careful assessment of potential immunogenicity, as this aspect could affect safety (especially when repeated dosing is required).

### 8.3. Scenario 3

If the pharmacokinetic (PK) profile differs due to sequence variations, then the PK data can be used to guide dose adjustments to achieve the desired clinical response. Sequence differences—especially in untranslated regions (UTRs) or codon optimization—can affect the stability and translation efficiency of mRNA and, thus, protein expression levels. Consequently, these variations can lead to differences in bioavailability and the duration of therapeutic protein exposure.

To achieve the same clinical response as the original product, the dose of the new mRNA product can be calculated through analyzing the PK parameters, such as peak concentration (Cmax), time to peak concentration (Tmax), area under the curve (AUC), and half-life. By understanding these parameters, the dose can be optimized to match the original product’s target therapeutic window and exposure profile. Key considerations include the following:

- Dose adjustment based on AUC: If the AUC differs, adjusting the dose to match the original product’s AUC can help to achieve comparable exposure over time, potentially leading to similar clinical efficacy.
- Peak and duration adjustments: Differences in C_max or half-life may require adjustment of the frequency or amount of dosing; for instance, if the modified mRNA degrades faster, more frequent or higher doses might be necessary to maintain therapeutic levels.
- Fine-tuning with therapeutic drug monitoring: If achieving an exact match is challenging, therapeutic drug monitoring (TDM) could be employed for individualized dosing in response to observed PK variability, ensuring that patients reach the intended therapeutic range.

A PK-based dose adjustment can help to compensate for sequence-related differences in mRNA stability or translation efficiency, ultimately enabling the new product to achieve a clinical effect that is comparable to that of the original. However, clinical response monitoring would still be necessary, mainly if the modified mRNA sequence introduces unforeseen differences in protein expression dynamics.

### 8.4. Scenario 4

The approach to comparing the toxicology of two mRNA products with similar or identical sequences depends on whether the LNP formulation is the same or differs between the two products. Optimal strategies for both cases are presented in the following:

### 8.5. Toxicology Comparison with Identical LNP Formulation

- In vitro assays: Start with in vitro toxicity assays to assess cellular toxicity and immune responses. Cytotoxicity, cellular uptake, and inflammatory marker assays (e.g., cytokine release profiles) can highlight any subtle differences in toxicity due to minor sequence variations.
- Acute and chronic toxicity studies: Conduct acute and chronic toxicity studies in animal models to observe any short term or long term toxicological impacts. Monitor biomarkers, organ weights, and histopathology to identify potential differences.
- Biodistribution and target organ toxicity: With identical LNPs, differences in biodistribution are less likely; however, biodistribution studies using labeled mRNA or LNPs can be conducted for confirmation. Focus should be placed mainly on toxicity in target organs (e.g., the liver and spleen, where LNPs tend to accumulate) to assess any subtle toxicological effects.
- Comparative immune response: After administration, assess immune activation markers (e.g., interferons, cytokines). Even with identical LNPs, slight mRNA sequence differences can alter the intensity or duration of the immune response.

### 8.6. Toxicology Comparison with Different LNP Formulations

- Comparative PK and PD studies: Begin with PK and pharmacodynamic (PD) comparisons in order to understand how differences in LNP formulations affect the biodistribution of mRNA and protein expression. Different LNP formulations may alter tissue targeting, clearance rates, and protein exposure levels.
- Immune response profiling: Different LNP formulations will likely elicit varied immune responses. If applicable, use immune profiling assays to measure systemic immune markers, such as cytokines (IL-6, TNF-α) and adaptive immune responses. This will help to assess any LNP-induced immunogenicity.
- In vivo toxicology studies with emphasis on LNP-sensitive organs: In vivo studies should assess organs where LNPs commonly accumulate (e.g., liver, spleen, lymph nodes). Detailed histopathological analysis and liver enzyme assays (e.g., ALT, AST) are critical for the detection of potential hepatotoxicity or immune cell infiltration in these organs.
- Comparative inflammatory and complement activation studies: Different LNP formulations can, to varying degrees, activate the complement system or inflammatory pathways. In vivo and ex vivo assays, such as the hemolysis assay for complement activation and inflammatory biomarker panels, will help to detect LNP-specific immune and inflammatory responses.
- Repeat-dose toxicity studies: Conduct repeat-dose toxicity studies in relevant animal models to evaluate cumulative toxic effects, which may be more pronounced with different LNP formulations. Monitoring clinical signs, body weight, hematology, and organ function over time will help to identify chronic toxicity risks.

Regulatory agencies will likely require comprehensive in vivo studies in both scenarios—even if the sequences are identical—due to possible differences in the behavior of and immune responses to LNPs. Moreover, any observed immunogenicity or toxicity differences must be carefully interpreted, especially for products intended for repeated administration. A stepwise approach, from in vitro to advance in vivo studies, offers the most robust comparison for assessing the safety and potential risks of each product.

To develop comprehensive guidelines for evaluating the similarity of mRNA products as biosimilars, it is essential to incorporate standards from biosimilar guidelines while addressing the unique structural, functional, and stability requirements specific to mRNA therapeutics. In this context, the guidelines must integrate aspects of molecular structure, stability, functionality, and immunogenicity for confirmation of equivalency with a reference mRNA product. A coherent, stepwise approach is outlined as follows.

### 8.7. Manufacturing Quality and Consistency

The foundation of comparability begins with stringent control over the manufacturing process. Quality by Design (QbD) principles, as defined in biosimilar guidelines, are fundamental to ensure that the manufacturing process yields consistent, high-quality mRNA with precisely controlled sequence, structure, and nucleotide modifications. This process includes stringent monitoring of critical process parameters, such as enzyme concentrations and nucleotide levels, during the in vitro transcription (IVT) phase, as this phase determines the sequence fidelity and modification consistency. The DNA template must be verified for quality, as even slight variations in the template could introduce sequence inconsistencies. When using modified nucleotides (e.g., pseudouridine), it is critical to validate their incorporation accurately, as modifications influence the stability and immunogenicity of the resulting products [89,90].

For mRNA products encapsulated in lipid nanoparticles (LNPs), maintaining consistency in particle size, encapsulation efficiency, and uniformity is essential, as these factors directly impact the efficiency of delivery. Good Manufacturing Practices (GMP) are also mandatory to prevent contamination and degradation, especially given the susceptibility of RNA to hydrolysis and oxidation [91].

### 8.8. Structural Characterization

Comparative structural analysis is fundamental to demonstrate that an mRNA biosimilar is identical to the reference product. Sequence identity, verified through high-throughput RNA sequencing, ensures that the nucleotide composition is exact, encompassing the coding sequence, untranslated regions (UTRs), and the poly(A) tail. Secondary structures formed by intramolecular base pairing—such as hairpins—must also be consistent, as these structures affect translation efficiency. Structural analysis using Selective 2′-Hydroxyl Acylation Analyzed by Primer Extension (SHAPE) or other probing techniques can help to map these secondary structures.

In addition, chemical characterization must confirm the incorporation of modified nucleotides, as these affect stability and immunogenicity. Mass spectrometry and high-performance liquid chromatography (HPLC) can quantify modified nucleotide ratios and their precise positioning within the mRNA. This step is necessary as slight modification differences could lead to immune recognition or altered translation dynamics [90].

### 8.9. Stability Testing

Stability assessments provide insights into the durability and resilience of mRNA products under various storage and handling conditions. Both accelerated and real-time stability tests, derived from biosimilar standards, should be conducted in order to simulate long term storage (e.g., −20 °C) and accelerated conditions (e.g., 40 °C with elevated humidity). Periodic sampling allows for the detection of degradation products, loss of potency, and other changes in product integrity over time.

Forced degradation studies under extreme pH, oxidative, and thermal conditions offer a comparative view of degradation pathways. Analytical tools such as HPLC, capillary electrophoresis, and UV spectroscopy can reveal physical and chemical degradation profiles. If the mRNA is encapsulated in LNPs, dynamic light scattering (DLS) and electron microscopy can track particle size changes and LNP stability under stress conditions.

Additionally, handling stability tests, including repeated freezing and thawing cycles, can be performed to assess whether the product retains its stability during routine clinical handling. This testing is especially relevant for mRNA, as freeze–thaw cycles can disrupt the integrity of RNA and the LNP structure, impacting the efficiency of delivery and therapeutic effectiveness.

### 8.10. Functional Testing

Functional testing is critical for establishing that the mRNA product translates into the intended protein at levels consistent with the reference. In vitro translation assays evaluate the protein yield, confirming that the mRNA efficiently directs protein synthesis in a controlled system. For more biologically relevant data, in vivo pharmacokinetic (PK) studies can be conducted to assess protein expression over time in an animal model, providing data on absorption, distribution, metabolism, and excretion (ADME) profiles. These PK studies reveal whether the mRNA product maintains bioavailability comparable to that of the reference mRNA.

Bioactivity assays can be conducted to further assess the functionality of the expressed protein. Cell-based or biochemical assays ensure that the protein maintains its intended biological activity, which is particularly important for therapeutic applications requiring specific binding or enzymatic action. This functional confirmation is necessary to ensure that the translated protein mirrors the therapeutic effects of the reference product.

### 8.11. Immunogenicity Assessment

Immunogenicity concerns are central to evaluating the safety of mRNA therapeutics. As immune responses can stem from both the mRNA and the LNPs used for encapsulation, a dual approach to immunogenicity testing is necessary. Assessing innate immune activation through cytokine release or toll-like receptor (TLR) assays provides insights into the initial immune response triggered by the mRNA product. This is especially pertinent if the mRNA contains unmodified nucleotides, which could be recognized as foreign by the immune system.

Assessment of the adaptive immune response, including the potential for anti-drug antibodies (ADA) against the expressed protein, requires testing in preclinical models and, ultimately, human studies. These evaluations serve to determine whether repeated administrations will lead to neutralizing antibodies that could reduce the efficacy of the drug or cause adverse effects [92].

### 8.12. Comparative Analytical Testing and Documentation

Comprehensive analytical testing must involve a comparison of the critical attributes of the mRNA product and the reference, ensuring their equivalency across purity, structural integrity, and bioactivity aspects. Both capillary and gel electrophoresis can be performed to assess RNA integrity, while HPLC and mass spectrometry serve to quantify impurities, such as truncated or double-stranded RNA byproducts.

The 5′ cap structure and poly(A) tail length are essential for stability and translation initiation. Reverse transcription polymerase chain reaction (RT-PCR) and enzymatic assays can be performed to confirm that these features align with those of the reference product. Furthermore, mass and charge distribution analyses through the use of advanced analytical methods ensure that the mRNA’s molecular profile is consistent, eliminating structural discrepancies.

Documentation consolidating all data from each phase of testing should be prepared, including process validation records, stability data, bioactivity results, and immunogenicity profiles. Detailed documentation provides a comprehensive reference for regulatory submission, allowing agencies to verify the equivalency claims.

## 9. Proposed Guideline

Creating a comprehensive guideline for mRNA products through the integration of relevant elements from biosimilar guidelines involves several key areas: manufacturing quality, stability, structural characterization, functional assessment, and immunogenicity. An outline of guidelines for mRNA therapeutics is presented below, tailored to meet their unique characteristics while drawing on established biosimilar principles (Table 6).

### 9.1. Quality by Design (QbD) and Manufacturing Controls

- Process development and control: Following a QbD approach, ensure that the manufacturing process is consistent and capable of producing high-quality mRNA with controlled sequence, structure, and modifications. Key aspects include the following:
- Template DNA quality: DNA template purity is essential, as it determines mRNA sequence fidelity.
- In vitro transcription (IVT) consistency: Controls on enzyme concentrations, nucleotide substrates, and buffer systems to achieve consistent transcription.
- Modified nucleotides: Validation is required to confirm the precise incorporation of modified nucleotides (e.g., pseudouridine), if they are used.
- Good manufacturing practices (GMP): Ensure GMP compliance, including aseptic processing for sterility and safety. This encompasses the monitoring of environmental conditions to protect RNA from degradation.
- Lipid nanoparticle (LNP) encapsulation: If using LNPs, specify the particle size, encapsulation efficiency, and homogeneity.

### 9.2. Structural Characterization

- Sequence identity: Use high-throughput RNA sequencing to verify that the mRNA sequence matches the reference sequence.
- Secondary structure verification: Secondary structures (e.g., hairpins) can influence translation efficiency and stability. Techniques such as Selective 2′-Hydroxyl Acylation analyzed by Primer Extension (SHAPE) or cryo-electron microscopy can be used to validate these structures.
- Modified nucleotide analysis: The presence, location, and ratio of modified nucleotides should be assessed using mass spectrometry or high-performance liquid chromatography (HPLC) to confirm that the reference profile is matched.

### 9.3. Stability Testing

- Accelerated and real-time stability: Conduct stability studies under accelerated (e.g., 40 °C, 75% RH) and real-time storage conditions in order to define the shelf-life of the product. Real-time studies are essential to simulate actual storage conditions (e.g., −20 °C).
- Thermal cycling studies: Test stability under repeated freezing and thawing cycles to mimic standard handling practices.
- Forced degradation studies: Subject the mRNA to stress conditions, such as extreme pH, oxidative stress, and light exposure, to identify degradation pathways and establish degradation products.
- LNP stability: For mRNA in LNPs, monitor the particle size and zeta potential over time. Use dynamic light scattering (DLS) or transmission electron microscopy (TEM) for LNP integrity analysis.

### 9.4. Functional Testing

- In vitro translation efficiency: Conduct in vitro translation assays to ensure that the mRNA product consistently translates into the desired protein with expected yield and activity.
- In vivo protein expression and PK studies: Perform animal studies to compare in vivo protein expression levels and pharmacokinetics (PK) with the reference mRNA product. This should include protein quantification, as well as assessments of its distribution, metabolism, and excretion.
- Bioactivity of expressed protein: Confirm that the protein expressed from the mRNA has comparable bioactivity to the reference product, using cell-based or biochemical assays for assessment of therapeutic efficacy.

### 9.5. Immunogenicity Assessment

- Innate immune activation: Assess the immune activation potential, especially if using unmodified mRNA. Testing can involve cytokine release assays or toll-like receptor (TLR) assays to evaluate the activation of the innate immune response.
- Adaptive immune response: Evaluate the immunogenicity of the expressed protein, primarily if the therapeutic mRNA is intended for repeated administration. Animal studies and in vitro assays can provide preliminary data, although clinical testing is ultimately required.
- Anti-mRNA antibody formation: If patients receive multiple doses, assess the risk of anti-drug antibodies (ADA) against the mRNA or the lipid nanoparticles, as these could impact the efficacy or safety of the product.

### 9.6. Comparative Analytical Testing (Drawing from Biosimilar Guidelines)

- Comparability protocol: Conduct a side-by-side analytical comparison with the reference product, covering the following:
- Purity and impurities: Capillary electrophoresis, HPLC, and other analytical methods can be used to quantify impurities, such as truncated RNA and double-stranded RNA by-products.
- 5′ cap and 3′ poly(A) tail length: Confirm that the mRNA 5′ cap structure and poly(A) tail length are consistent with the reference, as these impact the initiation of translation and stability.
- Mass and charge profile: Analyze the molecular mass and charge distribution to detect any variations in mRNA composition.

### 9.7. Additional Clinical Studies for Safety and Efficacy

- Pharmacodynamics (PD) and PK matching: Conduct clinical PK and PD studies in human volunteers to confirm that the mRNA product has bioavailability and therapeutic efficacy similar to those of the reference.
- Comparative safety assessment: Carry out single-dose and repeated administration safety assessments, especially for monitoring differences in immune response.

### 9.8. Documentation and Regulatory Compliance

- Detailed documentation: Compile all testing data, including batch records, stability reports, and clinical trial data, in accordance with regulatory requirements.
- Risk management: Follow risk assessment protocols to identify and mitigate potential risks unique to mRNAs, such as enzymatic degradation or immune activation.

This guideline provides a comprehensive approach to establishing the equivalency of mRNA products, drawing on biosimilar principles while addressing mRNA’s unique aspects in terms of stability, structure, and function.

## 10. Naming

Regulatory agencies face difficulties in naming products that are biologically similar to reference mRNA products. Biosimilars must meet the following criteria: the same route of administration, the same active ingredient, and exact dosing. This definition will be met for a product that is a copy of an approved mRNA product, except for the requirement of the same active ingredient. However, the active ingredient is delivered through a middle-delivery scheme in this case.

If an mRNA product is designed to replace a recombinant product, the definition of biosimilar is not met. This requires the creation of another category—likely a “hybrid biosimilar”—where equivalence is established via PK profile similarity, as the measured active molecule is the same. In many cases, such PK profile similarity will not be achieved, and so, the use of a pharmacodynamic marker may be helpful. Still, if there is no such definitive marker, clinical efficacy testing may be required. It is worth noting that biological drugs have broad dose–response relationships, and so, the clinical responses will likely be matched. These concerns need to be discussed and brought under the FDA GASK guideline that promotes their application. Scientific knowledge allows the developers to suggest regulatory compliance regardless of existing guidelines [87]. It is anticipated that the path for the introduction of new technologies will become easier and faster, as developers will be allowed to submit suggestions justifying the introduction of new technologies.

However, the FDA has confirmed to the author that, if the end-product of an mRNA product is well defined, it can be named as follows: Name of biological entity, mRNA (xxxx), injection.

## 11. Intellectual Property

According to the Derwent Global Patent Data (Clarivate), a total of 1213 patents related to mRNA-based medicines (including therapies, vaccines, and delivery systems) were filed worldwide from 2020 to April 2024. This activity represents an estimated 5.4-fold increase compared to the period from 2011 to December 2019, before the COVID-19 epidemic began [93].

While natural proteins are not patentable, modified or engineered protein variants that enhance specific properties—such as their stability or function—can be patented. For example, modified versions of sirtuins or Klotho proteins with amino acid changes may qualify for patent protection. Moreover, the therapeutic use of mRNA—for instance, using mRNA to produce proteins with anti-aging or health-promoting benefits—can be patented as a treatment method; for example, a method-of-use patent could cover the application of mRNA-encoding proteins such as sirtuin or Klotho to combat age-related cellular damage or improve metabolic health. New combinations of mRNA encoding multiple proteins (e.g., an anti-aging “cocktail” delivering SIRT1, Klotho, and AMPK) may also be patentable as a novel therapeutic approach. Moderna and BioNTech’s success with mRNA-based COVID-19 vaccines has illustrated how patents protect unique mRNA sequences, formulations, and delivery systems, and not the proteins themselves.

Similarly, patents cover the mRNA and delivery methods for anti-aging or other therapeutic proteins, rather than the natural proteins they produce. Thus, mRNA designed to deliver therapeutic proteins and specific delivery technologies and therapeutic applications are patentable. This allows companies to secure intellectual property rights over innovative mRNA-based therapies, even if the proteins they encode occur naturally.

As the landscape of patents on the products proposed is likely to expand rapidly, an understanding of the laws is essential for developers. Natural proteins, as they exist in the body, cannot be patented under U.S. patent law and similar laws worldwide, as they are considered “products of nature”. However, mRNA sequences designed to deliver or produce these proteins in the body can be patented for several reasons.

First, synthetic mRNA is regarded as an invention: designing, synthesizing, and modifying mRNA to deliver specific protein instructions safely and effectively in the body requires human ingenuity, making it a patentable invention. Modifications such as stabilization, codon optimization, or lipid nanoparticle encapsulation represent unique technical advances that are eligible for patenting.

Second, the delivery technology itself is patentable, including methods for delivering mRNA to specific tissues or cells and carriers such as lipid nanoparticles that transport the mRNA, which are essential for therapeutic effectiveness, especially in applications where targeted delivery is critical.

One primary intellectual property consideration is the LNP composition. Only three LNP-based products have been approved by the FDA.

- Patisiran (Onpattro) Approval Year: 2018. Treatment of polyneuropathy caused by hereditary transthyretin-mediated amyloidosis (hATTR). LNPs are utilized to deliver small interfering RNA (siRNA) targeting the transthyretin (TTR) gene, reducing production of the TTR protein.
- Pfizer-BioNTech’s Comirnaty and Moderna’s Spikevax: Approval Year: 2020. Indication: Prevention of COVID-19. LNPs are employed to encapsulate messenger RNA (mRNA) encoding the SARS-CoV-2 spike protein, facilitating cellular uptake and subsequent immune response.
- Moderna’s RSV Vaccine (mRESVIA): Approval Year: 202. Prevention of respiratory syncytial virus (RSV) in adults aged 60 and older. LNPs are used to deliver mRNA encoding RSV antigens, stimulating an immune response against the virus.
- Lipid nanoparticle (LNP) formulations are widely used for the delivery of therapeutic agents, particularly nucleic acids such as mRNA and siRNA. While patents protect many LNP technologies, some formulations are no longer under patent protection and are considered patent-free. These include early-generation LNPs and particular naturally occurring lipid-based delivery systems.
- Early LNP formulations, developed in the 1990s and early 2000s, often utilized cationic lipids such as DOTMA (N-[1-(2,3-dioleyloxy)propyl]-N, N,N-trimethylammonium chloride) and DOPE (dioleoylphosphatidylethanolamine). Many of these early patents have expired, rendering these formulations patent-free.

Certain lipid-based delivery systems, such as those using naturally occurring lipids without proprietary modifications, may not be covered by active patents; however, their efficacy and stability might be limited compared to more advanced, patented LNP technologies. This lower efficiency is significant for vaccines, but not as much when used to express functional proteins. Other formulations with expired patents utilized in FDA-approved products can be considered as a great choice; for example, the original lipid nanoparticle (LNP) formulation used by Onpattro (patisiran) during its development and early stages involved a specific combination of lipids optimized to deliver small interfering RNA (siRNA) efficiently. The primary components included the following:

- Ionizable lipid: DLin-MC3-DMA was the critical ionizable lipid in Onpattro’s formulation, which is specifically designed to become positively charged in the acidic environment of endosomes, aiding in the release of siRNA into the cell’s cytoplasm.
- Phospholipid: DSPC (1,2-distearoyl-sn-glycero-3-phosphocholine) is a phospholipid that provides structural stability to the lipid nanoparticle and supports its interaction with cell membranes.
- Cholesterol enhances the fluidity and stability of the LNP, improving its integrity and ability to circulate in the bloodstream.
- Polyethylene glycol (PEG) lipid: DMG-PEG2000 (1,2-dimyristoyl-rac-glycero-3-methoxy polyethylene glycol-2000).

The U.S. Patent No. 8,450,239, granted on 28 May 2013, covers the current composition of lipids used in Onpattro, outlining the ratios and characteristics of lipids, such as DLin-MC3-DMA, DSPC, cholesterol, and PEG-lipids, which allow for efficient nucleic acid delivery. This patent is expected to expire on 5 August 2028.

## 12. Conclusions

Recombinant technology made it possible to access proteins that control most bodily functions. Still, this technology comes at a high cost, making protein drugs—including functional proteins, engineered proteins, monoclonal antibodies, and protein vaccines—out of reach for most patients worldwide. Furthermore, even the development cycle for biosimilars is long and expensive, due to the very nature of the products in terms of their structural variability; this is one reason why only 16 out of more than 250 molecules are available as biosimilars. At present, the chemical-based mRNA technology is substantially less expensive to develop and manufacture; additionally, it poses fewer risks of side effects. mRNA technology is expected to allow for the entry of hundreds of protein drugs that could not be brought to patients due to the high development cost and low market potential of such drugs; furthermore, the hundreds of approved recombinant proteins could be transformed using mRNA technology, thus broadening their accessibility. Finally, approved mRNA products can be copied with much better assurance than the biosimilar copies of recombinant proteins, due to the chemical nature of the mRNA products. However, the acceptance of mRNA for protein drugs will require a significant shift in the thinking of regulatory agencies, who are generally slow to adopt novel pathways. However, the technical details and arguments presented in this paper may serve as a foundation for this planning. mRNA technology is here to stay, and, in many ways, recombinant technology can be expected to slowly wane out—how fast it happens will depend on the developers, who should capitalize on the FDA GASK guideline [87] to make proposals to the agencies for novel procedures that establish the comparable clinical efficacy of their products based on approved mRNA products or utilizing new mRNA delivery systems for novel proteins.

It is anticipated that, within a decade, as newer products such as those based on mRNA technology enter the market, recombinant technology will be phased out for these applications; even now, as discussed above, an mRNA substitute to recombinant protein is available, and it may be possible that, in less than 5 years, biosimilars may start arriving as mRNA-based products.

When a new technology is introduced, there are always concerns regarding how patients and prescribers will adopt it; however, the FDA has developed extensive education programs that should take care of these concerns, and I do not foresee this to be an issue, particularly considering that the price of these products is expected to be substantially less than that of recombinant products.

## Figures and Tables

**Table 1:** Types of mRNA.

mRNA Type	Applications	Properties	Regulatory Status
Conventional mRNA [4]	Vaccines	Basic structure with 5′ cap, UTRs, poly(A) tail	Approved for COVID-19 vaccines
Modified mRNA [1]	Protein replacement, cancer vaccines	Chemically modified nucleosides	Clinical trials for genetic diseases, COVID-19 vaccines
Self-amplifying mRNA (saRNA) [5]	Vaccines, gene therapies	Self-replicating sequences for dose-sparing	Early clinical trials
Circular RNA (circRNA) [6]	Long term protein replacement therapies	Circular structure for enhanced stability	Preclinical and early clinical stages
Therapeutic Guide RNA [7]	Gene editing for genetic disorders	Guide sequence directs gene editing	Clinical trials under ATMP designation

Note: As of November 2024, there were 785 clinical trials listed in clinicaltrials.gov [8].

**Table 2:** Animal toxicology testing plan.

Animal Species	Mouse
Duration in-life	28 days to 3 months
Administration	Repeated, subcutaneous
Dosage level	2
Groups	2: saline control, dose group
Group size	20
Total Animals	40
Monitoring	Mortality, body weight, clinical observation, food consumption, local reaction, body temperature, FOB (mod. Irwin), hematology, clinical chemistry, urinalysis, bone marrow smear, blood coagulation parameters, cytokines TNF-alpha, IFN-gamma, IL-10, etc.
Postmortem	Necropsy and weight of selected organs; histopathological evaluation, RNA biodistribution
Duration	3 months

**Table 4:** Characterization and release testing methods for mRNA drug substances.

Quality	Attribute	Method(s)
Identity	mRNA sequence identity confirmation	Sanger sequencing
Content	RNA concentration	Ultraviolet Spectroscopy (UV)
Integrity	mRNA intactness	Capillary electrophoresis
Purity	5′ capping efficiency	Reverse-phase liquid chromatography-mass spectroscopy (RP-LC-MS-MS)
	3′ poly (A) tail length	Capillary Electrophoresis
	Product-related impurities—dsRNA	Slot-blot
	Product-related impurities—aggregate quantitation	Size exclusion high-performance liquid chromatography (SE-HPLC)
	Process-related impurities—residual DNA template	Quantitative PCR (qPCR)
	Process-related impurities—quantitation of free/non-incorporated nucleosides	Reverse-phase liquid chromatography-mass spectroscopy (RP-LC-MS-MS)
	Process-related impurities—residual T7 RNA polymerase content	Enzyme-linked immunosorbent assay (ELISA)
Potency	Expression of target protein	Cell-based assay
Safety	Endotoxin	USP <85>
	Bioburden	USP <61>
Other	Appearance	USP <790>
	Residual solvents	USP <467>
	pH	USP <791>

**Table 5:** Drug product release specifications.

Quality	Attribute	Method(s)
Identity	RNA identification	Sanger sequencing
	Identity of lipids	Reversed-phase high-performance liquid chromatography with charged aerosol detector (RP-HPLC-CAD)
Content	RNA concentration/RNA encapsulation efficiency	Ribogreen Assay
	Lipid content	Reversed-phase high-performance liquid chromatography with charged aerosol detector (RP-HPLC-CAD)
Integrity	LNP size and polydispersity	Dynamic light scattering (DLS)
	RNA size and integrity	Capillary electrophoresis (CE)
Potency	Expression of target protein	Cell-based assay
Purity	Product-related impurities—aggregate quantitation	Size exclusion high-performance liquid chromatography (SE-HPLC)
	Product-related impurities—the percentage of fragment mRNA	Ion-pair reversed-phase high-performance liquid chromatography (IP-RP-HPLC)
Safety	Endotoxin	USP <85>
	Sterility	USP <71>
Other	Appearance	USP <790>
	pH	USP <791>
	Subvisible particles	USP <787>
	Osmolality	USP <785>
	Residual solvents	USP <467>
	Extractable volume	USP <1>, USP <698>
	Container closure integrity	USP <1207>

**Table 6:** Summary of key comparability requirements (adapted from biosimilar guidelines).

Testing Category	mRNA-Specific Considerations	Biosimilar Guideline Parallels
Sequence identity	Complete sequence and modified nucleotides	Amino acid sequence identity
Secondary structure	SHAPE and cryo-EM for folding patterns	Protein folding and glycosylation
Stability testing	Thermal cycling, forced degradation	Real-time, accelerated, forced degradation
Functional testing	In vitro translation, in vivo protein expression	Cell-based assays for protein activity
Immunogenicity	Innate and adaptive response assessments	Anti-drug antibody and immune response testing
PK/PD clinical testing	Protein expression PK and therapeutic bioactivity	PK/PD comparison in clinical studies
